# Immune response impairs learning in free-flying bumble-bees

**DOI:** 10.1098/rsbl.2008.0331

**Published:** 2008-07-15

**Authors:** A Alghamdi, L Dalton, A Phillis, E Rosato, E.B Mallon

**Affiliations:** 1 Biology Department, University of LeicesterLeicester LE1 7RH, UK; 2 Genetics Department, University of LeicesterLeicester LE1 7RH, UK

**Keywords:** psychoneuroimmunology, cross-talk, social insects

## Abstract

Parasites can influence different host behaviours including foraging, mate choice and predator avoidance. Several recent papers have shown reduced learning abilities in infected insects. However, it is difficult to separate the effects of the immune response from the direct effects of the parasite. Using a free-flying learning paradigm, this paper shows that learning performance is impaired in bumble-bees (*Bombus terrestris*) that are not infected but whose immune system is stimulated non-pathogenically. This demonstrates that before it is assumed that a parasite has a direct effect on a host's behaviour, the effect of the immune response stimulated by the parasite must first be quantified.

## 1. Introduction

Parasites can influence different host behaviours including foraging, mate choice and predator avoidance (Moore 2002). Several recent papers have shown reduced cognitive abilities in infected insects (Gegear *et al*. 2006; Iqbal & Mueller 2007). In nature, this would have severe fitness costs (Raine & Chittka 2008). However, although parasites act directly on hosts, a growing number of general pathologies have been shown to be the result of the immune response elicited by the parasite (Moret & Schmid-Hempel 2000). Bumble-bees (*Bombus impatiens*) infected by a protozoan parasite (*Crithidia bombi*) have an impaired ability to learn the colour of rewarding flowers (Gegear *et al*. 2006). Although this infection-induced change to cognitive function in bumble-bees could be the direct effect of the parasite, this seems unlikely as *Crithidia* is restricted to the gut of the bumble-bee (Gegear *et al*. 2006). It seems more likely that this behaviour is caused by the actions of the immune response.

There is extensive communication between the central nervous system and the immune system (Dantzer 2004). Many behavioural responses to infectious agents, such as fever, increased slow-wave sleep, reduced activity, exploration and sexual behaviour in mammals, are orchestrated by immune products called proinflammatory cytokines that are released in response to the detection of antigens (Maier & Watkins 1998). Links between the nervous and immune systems are not unique to vertebrates. We have shown that both the honeybee *Apis mellifera* (Mallon *et al*. 2003*a*) and the bumble-bee *Bombus terrestris* (Riddell & Mallon 2006) perform poorly in proboscis extension reflex (PER) memory tests (Bitterman *et al*. 1983) when their immune systems have been challenged by lipopolysaccharide (LPS). LPS is a component of Gram-negative bacterial cell walls, which is a non-pathogenic elicitor of the immune response (Moret & Schmid-Hempel 2000). That is, we found that learning and memory are impaired by the immune response directly with no parasite present.

In this study, we use a free-flying floral choice assay to test the learning abilities of bumble-bees (*B. terrestris*). Instead of using a live parasite to affect cognitive function (Gegear *et al*. 2006), we use LPS. Gegear *et al*. proposed that the *Crithidia*-induced alteration in memory abilities that they found is most likely caused by the host's own immune system, and not the direct action of the parasite itself. If the Gegear result is replicated in our study, it will show that this is indeed the case. It will also show that the previously found immune-induced memory reduction (Riddell & Mallon 2006) is reproducible in a free-flying semi-natural paradigm, providing evidence that this connection between the immune response and learning/memory in insects is a general and vitally important part of their ecology.

## 2. Material and methods

Experiments were carried out on two commercially reared bumble-bee colonies from Koppert Biological Systems, UK. The experiments began when the colonies had a minimum of 30 workers, approximately four weeks old. Between observations, the colonies were fed ad libitum with pollen (Percie du Sert, France) and 50% diluted glucose/fructose mixture (Meliose; Roquette, France). Before the experiments, the colonies were kept at 26°C and 60% humidity in constant red light.

### (a) Tagging and injection

Each individual bee was marked with a unique tag. We challenged the bee's immune system by injecting, into the haemolymph, a dose of 5 μl of Ringer's solution, containing 4% LPS (Sigma L-2755; 0.5 mg ml^−1^=9 mg 4% LPS g^−1^ of bee) that is a highly immunogenic but non-pathogenic elicitor of the immune response (Moret & Schmid-Hempel 2000). Workers in each colony were assigned randomly to either the treatment group where they were injected with LPS or to the control group where they were injected with 5 μl of Ringer's solution, a saline solution regularly used in insect physiology. As new workers eclosed, they were assigned alternatively to either the LPS or the control group. The bees were at least 5 days old when injected. Each bee was re-injected every 10 days to ensure their immune system remained stimulated (Korner & Schmid-Hempel 2004).

### (b) Learning assays

The bees were left for 4 days after injection before observations were begun to ensure that the immune system of LPS bees had been stimulated (Korner & Schmid-Hempel 2004). We connected the nest-box to the flight arena, a 1200×1000×300 mm plywood box with a removable Perspex lid. All observations were carried out at 23°C and 60% humidity with a 12 L : 12 D cycle. The reduced temperature discouraged the colony from moving into the flight arena. For each colony, we used a slightly different training method.

#### (i) Training method A

During training method A, used for colony 1, we put two Petri dishes (90 mm in diameter) in the centre of the arena, 100 mm apart. Each contained, randomly arranged, 13 yellow ‘flowers’ (inverted coloured standard screw tube caps: 11 mm in diameter and 5 mm in depth; Scientific Specialities, Inc.) and 13 ‘blue flowers’. They also contained seven green podiums (caps topside up) placed at regular intervals. The maximum distance between the flowers was a single podium diameter. During the pretraining phase, bees foraged freely from either flower, both of which contained 15 μl of 50% (w/w) sugar water. The bees completing at least five consecutive foraging bouts were chosen for training. During the training phase, each rewarding flower was filled with 15 μl sugar water (50% w/w) and the unrewarding flower was filled with 15 μl water to ensure that the bees could not discriminate by sight alone. Once a bee began foraging only it was allowed in and out of the flight arena. We recorded the colour of the flowers visited for a total of 90 successive visits for each bee. Once a bee temporarily left the flight arena, the flowers were refilled manually with a pipette. After each individual bee's visits, the flight arena was cleaned using 70% industrial methylated spirit. The colour of the rewarding flower was alternated between the bees. It was noted that the bees often walked between the flowers in the same Petri dish.

#### (ii) Training method B

Training method B, which was used for colony 2, is similar to the method outlined in Raine *et al*. (2006). We found this to be a simpler way of recording free-flying learning ability, specifically the bees were required to land on each flower and could not walk rapidly between the flowers. During the pretraining, the bees foraged from 20 randomly placed blue or yellow flowers, both containing 15 μl of sugar water. The flowers consisted of an inverted Eppendorf tube on a small platform (20 mm in diameter), 50 mm from the floor of the arena. The bees completing at least five consecutive foraging bouts were selected for training. Ten blue flowers and 10 yellow flowers were placed at random locations within the arena. The yellow flowers were always rewarding, as the bees show an innate preference for the blue flowers (Raine *et al*. 2006). For each bee, we recorded 89 flower choices after the bee first chose a rewarding flower. That is, each bee visited a minimum of 90 flowers.

### (c) Statistical analysis

The method of generalized estimating equations was used to account for correlations among observations from the same subject in a repeated measures logistic regression (Hardin & Hilbe 2003). The dependent variable was ‘visit to rewarding flower’ (yes/no). The independent variables were visit (1–90) and treatment (LPS or Ringer).

## 3. Results

We tested 31 bees injected with LPS (colony 1: 16 and colony 2: 15) and 27 injected with Ringer's solution (colony 1: 12 and colony 2: 15). As each colony was trained in a different way, each colony was analysed separately. The probability of both LPS and Ringer bees choosing the rewarding flowers increases over subsequent visits, i.e the bees learn (visit: colony 1 *z*=14.59, *n*=2520, *p*<0.0001 and colony 2 *z*=7.84, *n*=2690, *p*<0.0001; figure 1). However, we found that LPS bees take a longer time to learn the colour of the rewarding flowers (treatment: colony 1 *z*=−3.37, *n*=2520, *p*=0.001 and colony 2 *z*=−9.3, *n*=2690, *p*<0.0001; figure 1). This is especially obvious in the results of colony 2 where LPS bees have a much lower probability of choosing a rewarding flower in earlier visits when compared with the Ringer bees. In both colonies, figure 1 shows that by the eighth visit block, the proportion of the rewarding flowers is the same for both LPS and Ringer bees.

For colony 2, treatment (LPS versus Ringer) had no effect on how quickly the bees first found a rewarding flower (Mann–Whitney: *U*=−1.378, *n*=30, *p*=0.1681, median value=7 visits). This suggests that treatment does not affect the naive colour choice of bees. Owing to the design of training method A, this information is not available for colony 1.

## 4. Discussion

We found that bees whose immune systems were stimulated non-pathogenically by LPS had an impaired ability to learn the colour of the rewarding flowers. This result mirrored the effects found in both PER assays using LPS (Riddell & Mallon 2006) and the performance of bumble-bees in a flower choice assay when infected by *Crithidia* (Gegear *et al*. 2006).

Previous work has shown a correlation between the performance of honeybees in the PER assays and in the free-flying experiments (Laloi *et al*. 2000). One difference that we found between our PER work and our current study involves protein consumption. In both honeybees and bumble-bees, immune-induced PER impairment was only recorded when protein consumption was restricted (Riddell & Mallon 2006). From this, we suggested that increased protein consumption ameliorated the effects of the immune response on memory. However, in this current free-flying experiment, protein was not controlled, yet a clear effect was present. A recent study has shown that an immune response increases the food intake of bumble-bees (Tyler *et al*. 2006). It is also known that if a bumble-bee is allowed to forage, its immune response is decreased (Konig & Schmid-Hempel 1995). We hypothesize that during the PER assays, the harnessed bees have reduced protein requirements, therefore to expose the immune-induced reduction in memory we must control protein consumption. In the free-flying experiment, foraging as a physical act has a protein cost, therefore the protein requirements of the bee are increased. The bee then exhibits the immune-induced cognitive impairment without artificial external protein reduction.

Although both the experimental assays show a reduction in the ability to choose the correct flower type, the degree of immune-induced impairment in colony 2 (figure 1*b*) seems to be much stronger than in colony 1. It has been shown that bumble-bee colonies differ in both the level of their immune response (Mallon *et al*. 2003*b*) and their performance in associative learning tasks (Raine *et al*. 2006). It is possible that the colonies vary in how much their cognitive abilities are affected by their immune response. However, we feel this is a question for future work. Our two colonies' results cannot be directly compared as the assays used to test them were different. The larger impairment seen in colony 2 is still within the range of abilities found naturally in colonies (Raine & Chittka 2008).

This paper shows that the previously found decreased PER ability in immune-stimulated bees can be generalized to a more natural learning paradigm and a more realistic nutritional status. Recently, it has been shown that learning ability has a direct effect on bumble-bee colony fitness (Raine & Chittka 2008). This opens up the possibility that this cross-talk between the immune and the nervous systems could have vital fitness costs. Our results clearly demonstrate that before it is assumed that a parasite has a direct effect on a host's behaviour, the effect of the immune response stimulated by the parasite must first be quantified.

## Figures and Tables

**Figure 1:**
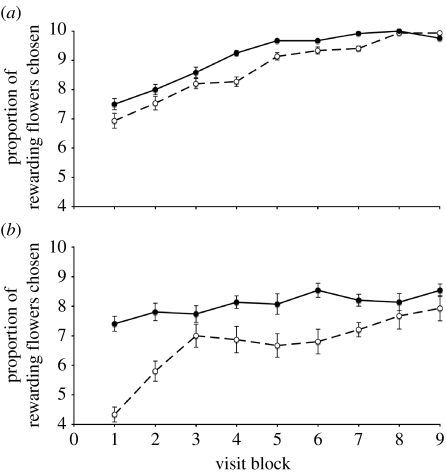
The proportion of rewarding flowers chosen by bees in 10 visit blocks. (*a*) Colony 1 and (*b*) colony 2. The open circles represent the means of bees injected with LPS. The filled circles represent the means of those injected with the Ringer control. Error bars represent the standard error.

## References

[bib1] Bitterman M.E, Menzel R, Fietz A, Schaefer S (1983). Classical conditioning of proboscis extension in honey bees. J. Comp. Physiol. Psychol.

[bib2] Dantzer R (2004). Innate immunity at the forefront of psychoneuroimmunology. Brain Behav. Immun.

[bib3] Gegear R.J, Otterstatter M.C, Thomson J.D (2006). Bumble-bee foragers infected by a gut parasite have an impaired ability to utilize floral information. Proc. R. Soc. B.

[bib4] Hardin J, Hilbe J.M (2003). Generalized estimating equations.

[bib5] Iqbal J, Mueller U (2007). Virus infection causes specific learning deficits in honeybee foragers. Proc. R. Soc. B.

[bib6] Konig C, Schmid-Hempel P (1995). Foraging activity and immunocompetence in workers of the bumble bee *Bombus terrestris* L. Proc. R. Soc. B.

[bib7] Korner P, Schmid-Hempel P (2004). *In vivo* dynamics of an immune response in the bumble bee *Bombus terrestris*. J. Invertebr. Pathol.

[bib8] Laloi D, Bailez O, Roger B, Pham-Delegue M.H, Wadhams L.J (2000). Recognition of complex odors by restrained and free-flying honeybees, *Apis mellifera*. J. Chem. Ecol.

[bib9] Maier S.F, Watkins L.R (1998). Cytokines for psychologists: implications of bi-directional immune to brain communication for understanding behaviour, mood, and cognition. Psychol. Rev.

[bib10] Mallon E.B, Brockmann A, Schmid-Hempel P (2003a). Immune response inhibits associative learning in insects. Proc. R. Soc. B.

[bib11] Mallon E.B, Loosli R, Schmid-Hempel P (2003b). Specific versus nonspecific immune defense in the bumblebee, *Bombus terrestris* L. Evolution.

[bib12] Moore J (2002). Parasites and the behaviour of animals.

[bib13] Moret Y, Schmid-Hempel P (2000). Survival for immunity: the price of immune system activation for bumblebee workers. Science.

[bib14] Raine N.E, Chittka L (2008). The correlation of learning speed and natural foraging success in bumble-bees. Proc. R. Soc. B.

[bib15] Raine N.E, Ings T.C, Ramos-Rodriguez O, Chittka L (2006). Intercolony variation in learning performance of a wild British bumblebee population (Hymenoptera: Apidae: *Bombus terrestris audax*). Entomologia Generalis.

[bib16] Riddell C.E, Mallon E.B (2006). Insect psychoneuroimmunology: immune response reduces learning in protein starved bumblebees (*Bombus terrestris*). Brain Behav. Immun.

[bib17] Tyler E.R, Adams S, Mallon E.B (2006). An immune response in the bumblebee, *Bombus terrestris* leads to increased food consumption. BMC Physiol.

